# Emergency therapeutic leukapheresis in a case of acute myeloid leukemia M5

**DOI:** 10.4103/0973-6247.39506

**Published:** 2008-01

**Authors:** Sudha Ranganathan, Shyamala Sesikeran, Vineet Gupta, Vanajakshi

**Affiliations:** *Apollo Hospital, Hyderabad*

**Keywords:** Emergency, hyperleukocytosis, leukapheresis

## Abstract

Cell separators in India are routinely used for plateletpheresis, peripheral blood stem cell collections and therapeutic plasma exchange. Therapeutic leukapheresis, particularly as an emergency procedure, has been uncommonly performed and reported. Here, a case of a 53-year-old male, diagnosed with acute myeloid leukemia subtype M5 (AML M5) with hyperleukocytosis, who underwent emergency leukaphereis, is reported. After two procedures, there was a decrease of WBC count by 85%, which enabled cytotoxic therapy to be initiated.

Therapeutic leukapheresis is performed in patients with hyperleukocytosis, a condition where WBC counts are more than 1,00,000/cu mm.[1] Hyperleukocytosis leads to leukostasis, especially in brain or lungs, which leads to cerebrovascular insufficiency or pulmonary leukostasis, respectively; the latter is characterized by dyspnea, tachypnea and hypoxemia without hypercapnea.[2] About 5-13% of adults diagnosed with acute myeloid leukemia present with hyperleukocytosis[3] and the risks of leukostasis appear to be greater in acute promyelomonocytic leukemia.[2] The morbidity and mortality associated with hyperleukocytosis forms the basis of performing leukapheresis in these patients. Guidelines published by the American Society For Apheresis and the American Association of Blood Banks state that therapeutic leukapheresis is indicated for hyperleukocytosis.[4]

## Case Report

A 53-year-old male weighing 56 kg was admitted with complaints of weakness since the last two weeks, fever and breathlessness for a week and haematuria for two days. On examination, he was pale, tachypnoeic with a respiratory rate of 40/minute and had purpuric spots over upper and lower limbs; generalized lymphadenopathy was absent. Abdominal examination showed distension but no organomegaly or free fluid. Both lungs showed normal air entry. Cardiovascular system was normal. The complete blood picture showed the following findings: Hb 11.4 gms%, hematocrit 30%, WBC 2,83,000/μl and platelet count 48,000/μL. The counts were performed on ABX Pentra 120 (Biomerieux, France), in which the maximum linearity for WBC was 2,00,000/μL. A one in two dilution in saline was performed to determine the actual WBC counts. ESR was 105 mm/hour. Peripheral smear showed monoblasts 29%, promonocytes 65%, myelocytes 2%, neutrophil 0%, marked immature leukocytosis with absolute neutropenia, thrombocytopenia and normochromic anemia. Prothrombin time (PT), activated partial thromboplastin time (aPTT) and International Normalized Ratio (INR) were within normal limits. Bone marrow examination showed a hypercellular marrow, decreased number of normal megakaryocytes, and normoblastic and diminished erythropoiesis. Granulopoiesis was abnormal with myelocytes 1%, promonocytes 35%, monoblasts 55% and myeloblasts 9%. The blasts showed fine diffuse PAS-positive granules and 23% of them were positive for peroxidase. AML M5 was diagnosed based on the above morphological features and was confirmed with a flow cytometric analysis. Chest X-Ray was normal. The patient was referred to the blood bank for emergency leukapheresis. Leukapheresis was performed on two consecutive days using Baxter CS 3000, using bilateral antecubital veins as the vascular access, after due consent from the patient. Seven and half liters of blood was processed on each day during the procedure.[2] ACD was used as the anticoagulant and the anticoagulant to blood ratio was maintained at 1:10. The blood flow was kept at 50 ml/minute. Details of the procedure, and pre and post apheresis counts are shown in Table 1. A total of 750 ml of ACD was used in each procedure. At the end of the second day's procedure, the WBC count was 46,000/cu mm, showing a reduction of 85% of WBC. There was a platelet reduction of 83%. No platelets were transfused because there was no symptomatic bleeding. Adverse effects in the form of citrate toxicity or vasovagal reactions were not encountered during or after the procedure. After the end of the first day's procedure and before the second day's procedure was started, the antecubital veins were flushed with 2 ml of Heplock (each ml of Heplock contains 10 USP heparin units, sodium chloride 0.9% w/v, benzyl alcohol 0.95% w/v). The patient was started on chemotherapy consisting of adriamycin and daunorubicin on the day after leukapheresis.

**Table 1:** Details of leukapheresis

Volume processed liters	Intervention	Pre-apheresis counts			Post-apheresis counts		
			
		Hb g%	WBC/µL	PLT/µL	Hb g%	WBC/µL	PLT/µL
7.5	Leukapheresis	10.9	2,83,000	48,000	9.8	2,38,000	34,000
7.5	Leukapheresis	9.8	2,40,000	12,000	6.6	46,000	8,000

WBC reduction 85%, Platelet (PLT) reduction 83%

## Discussion

In India, cell separators have routinely been used to collect platelets from normal healthy donors. Few centers additionally perform peripheral blood stem cell apheresis and therapeutic plasma exchange. Using cell separators for therapeutic and life-saving apheresis procedures in patients is very challenging. Here, a case of a patient who was referred to the blood bank for an emergency leukapheresis due to hyperleukocytosis with a WBC count of 2,83,000/μL is reported. Hyperleukocytosis was first described by Freirich *et al*.[5] and it has been reported that all patients with WBC counts of 2,00,000/μL or above showed either thrombi or cell aggregates.[2] In our case, though the patient had breathlessness, there were no other signs of hyperleukocytosis. Patients with hyperleukocytosis may present as a medical emergency requiring prompt recognition and initiation of therapy to prevent respiratory failure or intracranial hemorrhage.[6] Leukapheresis removes the circulating blasts quickly to alleviate symptoms whereas chemotherapy would take 24-48 hours to achieve the same effect.[7] Therapeutic leukapheresis has also been used prophylactically to reduce the risk of tumour lysis syndrome, which may occur in patients with leukemia with high blast counts. In these patients, the initiation of chemotherapy leads to rapid cell death and hyperuricemia resulting in acute renal failure.[8] Reduction of WBC counts after leukapheresis has been reported to be in the range of 15-46%[9] in one study and 50-86%[10] in another. A reduction of 85% in WBC counts was seen in the case presented here and this helped in the initiation of chemotherapy.[11] Emergency therapeutic leukapheresis for hyperleukocytosis is thus a safe procedure and helps in a rapid reduction of circulating leukocytes.
